# Spatio-temporal prediction of the COVID-19 pandemic in US counties: modeling with a deep LSTM neural network

**DOI:** 10.1038/s41598-021-01119-3

**Published:** 2021-11-05

**Authors:** Behnam Nikparvar, Md. Mokhlesur Rahman, Faizeh Hatami, Jean-Claude Thill

**Affiliations:** 1 The William States Lee College of Engineering, University of North Carolina at Charlotte, 9201 University City Blvd, Charlotte, NC 28223 USA; 2 Department of Urban and Regional Planning, Khulna University of Engineering & Technology (KUET), Khulna, 9203 Bangladesh; 3 Department of Geography and Earth Sciences, University of North Carolina at Charlotte, 9201 University City Blvd, Charlotte, NC 28223 USA; 4 School of Data Science, University of North Carolina at Charlotte, 9201 University City Blvd, Charlotte, NC 28223 USA

**Keywords:** Diseases, Infectious diseases, Population dynamics

## Abstract

Prediction of complex epidemiological systems such as COVID-19 is challenging on many grounds. Commonly used compartmental models struggle to handle an epidemiological process that evolves rapidly and is spatially heterogeneous. On the other hand, machine learning methods are limited at the beginning of the pandemics due to small data size for training. We propose a deep learning approach to predict future COVID-19 infection cases and deaths 1 to 4 weeks ahead at the fine granularity of US counties. The multi-variate Long Short-term Memory (LSTM) recurrent neural network is trained on multiple time series samples at the same time, including a mobility series. Results show that adding mobility as a variable and using multiple samples to train the network improve predictive performance both in terms of bias and of variance of the forecasts. We also show that the predicted results have similar accuracy and spatial patterns with a standard ensemble model used as benchmark. The model is attractive in many respects, including the fine geographic granularity of predictions and great predictive performance several weeks ahead. Furthermore, data requirement and computational intensity are reduced by substituting a single model to multiple models folded in an ensemble model.

## Introduction

The highly infectious COVID-19 pandemic that has been with us since the early months of 2020 has been adversely affecting public health worldwide with about 155 million reported cases of infection and 32 million deaths as of May 5, 2021^1–3^. Despite adopting non-pharmaceutical social distancing measures (e.g., travel ban, cancellation of flights, restrictions on gatherings, closure of schools and public transport) and pharmaceutical measures (i.e., vaccination), the number of coronavirus cases increased alarmingly fast in the United States and elsewhere throughout large periods of the pandemic^4–8^. Since March 2021, the COVID-19 pandemic has jumped globally towards a new peak exceeding the previous peak of January 2021 due to uncontrolled outbursts in India, Europe, and South America^3,5^. Considering the catastrophic consequences of this pandemic, this study envisioned to develop a purely data-driven model to forecast the number of infection cases and deaths 4 weeks ahead using a multi-variate deep long short-term memory (LSTM) network to guide policymakers to make timely appropriate decisions.

**Figure 1:**
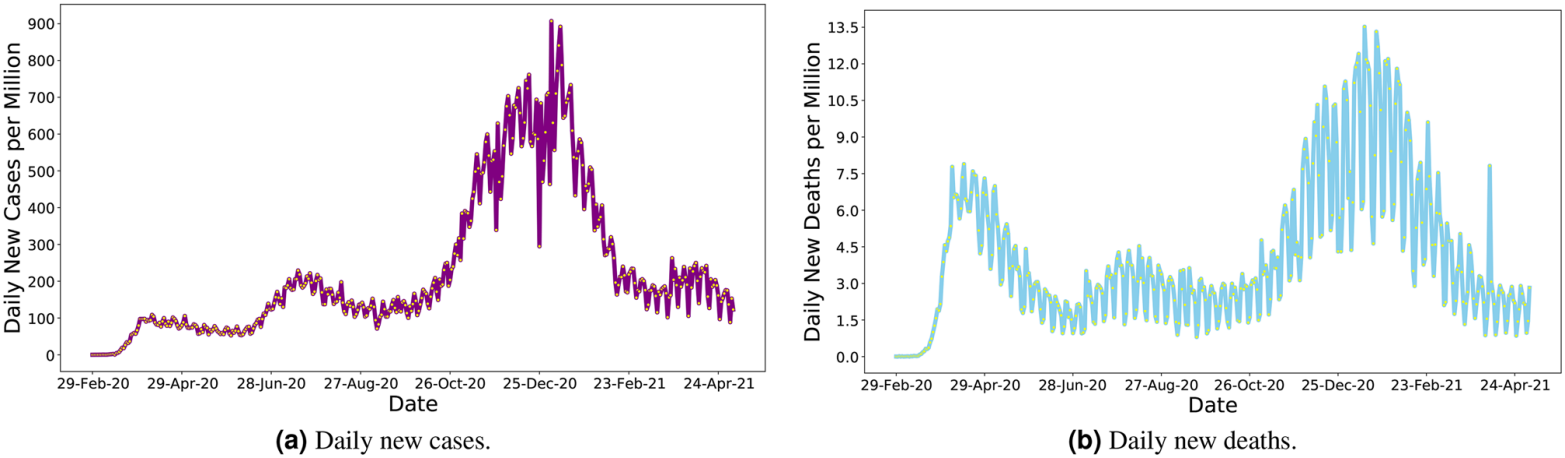
Daily new COVID-19 confirmed cases and deaths in the US, Data source^9^.

**Figure 2:**
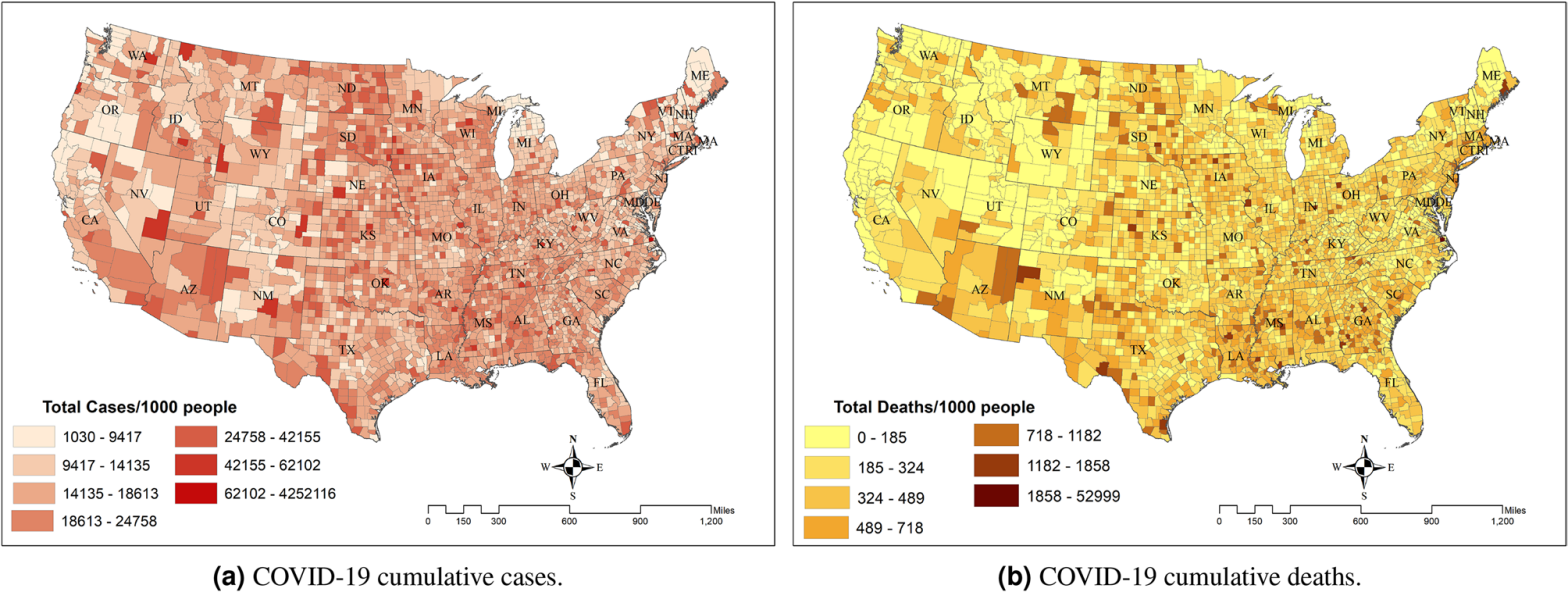
COVID-19 cases and deaths by county in the lower 48 United States, Data source^19^.

Although COVID-19 related infection cases and deaths have decreased by the time of this study, US citizens experienced the highest peak of the pandemic from November 2020 to February 2021 (Fig. 1). Despite strong pharmaceutical and non-pharmaceutical control measures, large cohorts of people have been affected daily in all states (Fig. 2). Well into this pandemic, the US has remained the most affected country in the world, with 21.39% and 18.23% of global confirmed cases and deaths, as of May 5, 2021^2^. However, the severity of the pandemic has started to taper off nationally since late February thanks to the successes of recent vaccine administration (i.e., 32% of people was fully vaccinated and 45% had at least one dose as of May 5, 2021)^3^. Moreover, multiple new variants of the COVID-19 virus that transmit more readily from person to person and change the effectiveness of the vaccines are emerging and circulating in the US and around the world^10^. Additionally, people are hesitant to receive vaccine against COVID-19 infection in the US^11^. Thus, a combination of evidence-based pharmaceutical and non-pharmaceutical public health mitigation measures remains important to curtail viral transmission locally and beyond^12–18^.

## Literature review

The extant literature shows that, from the inception of the pandemic, researchers from around the world have been developing and implementing COVID-19 prediction models to understand the severity of the pandemic, delineate associated factors of virus infection, recovery, and death, and support the design of effective policies and operational measures to manage this unprecedented public health crisis^1,15,20–22^. A wide range of methods and tools has been used in predictive models. Many studies have used machine learning and deep learning (e.g., random forest, support vector machine, decision tree, artificial neural network, ridge and lasso regression, nearest neighbor methods) to predict the transmission of the COVID-19 virus as well as individual and group responses to it^23–26^. A voluminous body of research has also been conducted with traditional epidemiological models to estimate viral diffusion and forecast the impact of policy interventions on the rate of infection (e.g., the Susceptible-exposed-infectious-removed (SEIR) model and variants of it)^27–31^. However, compartmental models share several critical limitations. Most importantly, they are dependent on a considerable number of hypothesized input parameters, including the probabilities for transitions of population between the S, E, I and R states. Because of the strong sensitivity of SEIR models to changes in these input parameters, predictive accuracy can be substantially downgraded. In addition, SEIR models are based on oversimplifying assumptions, one of which being that the probability values or transition rates are homogeneous over population^32^ and constant over time. Instead, the rates of transition between the S, E, I and R population compartments for COVID-19 change over time and are very sensitive to socio-demographic conditions and mitigation policies. To alleviate some of these drawbacks, other classes of models have been advanced for estimating the probability of COVID-19 transmission and identifying compliance with social distancing measures. These include various hybrid models integrating neural networks and SEIR modeling^33,34^, simulation systems (e.g., agent-based models)^35–37^ and video processing for object detection (e.g., You Only Look Once (YOLO))^38,39^. Some other studies have used econometric models (e.g., linear regression, structural equation model) to identify the factors that influence the diffusion of COVID-19 and associated mitigation measures^16,40^.

The convergence of recent developments in Information and Communication Technologies and in data analytics (i.e., production, access and storage of data, and analysis of information, cloud computing) has empowered people to apply Artificial Intelligence (AI) (e.g., machine and deep learning, text mining) to model and control complex issues in health care systems^41–43^. Hence, AI-based techniques are widely used to monitor social distancing patterns of people and to assess scenarios on the transition of COVID-19 accurately^15^. They can solve multi-scalar, endogenous, non-linear, and ambiguous problems and extract insights from complex, unstructured and large data sets using their computational ability with reliable prediction compared to traditional approaches. Additionally, hybrid models (i.e., combining of multiple machine learning techniques) may enhance the robustness and generalization ability of machine learning models to handle reputedly wicked problems, such as a viral pandemic, quickly and effectively compared to single machine learning techniques and simulation models^44,45^. One of the main motivations to predict the COVID-19 pandemic with machine learning is that it can more effectively estimate the effects of social distancing measures on the transmission of virus considering socio-economic control factors, which conventional econometric methods or compartmental models pain to capture^15^. However, high-quality data devoid of collection biases, robust methodologies, and validation with external data sets and models are necessary to develop a ubiquitous, reliable, and trustworthy model to provide consistent predictions across diverse systems (e.g., data, settlement contexts)^15,43,46^.

Considering the rapid transmission of COVID-19 and the time lapse for the implementation of control measures, many studies have conducted spatio-temporal disease modeling to understand the geographic exposure and evolution patterns of associated risks of the pandemic^4,47–51^. Spatio-temporal models are widely used by researchers to explain the temporal progress of disease over geographic regions and patterns of infection and mortality rates^52^. Recent studies have demonstrated that space and time are two critical factors for determining health risks during the COVID-19 pandemic^53,54^. Spatio-temporal models consider infection rates of the surrounding neighbors to reduce the variability in the estimation that may exist locally^52^. These models also consider retrospective infection rates for short- and long-term prediction, controlling the irregular reporting of daily confirmed cases and deaths. Thus, spatio-temporal models yield better prediction, with a higher prediction accuracy and a lower tendency to over- or under-fit compared to traditional epidemiological and machine learning-based models^47^. In this study, we used a deep learning-based space-time LSTM network to predict future COVID-19 cases and deaths, which can guide policymakers for timely interventions to reduce the severity of the pandemic and for more effective management of health care resources (e.g., nursing and medical staff, ICU beds, supplies, etc) under emergency conditions.

A wide range of studies have developed models for predicting COVID-19 cases and deaths and have investigated the factors that influence virus diffusion using machine learning and deep learning^15,21,55^. They mentioned that lockdown and confinement measures, and socioeconomic factors significantly influence the outbreak of the pandemic and its dynamics. Specifically, the family of deep recurrent neural networks have proven to be an attractive approach for epidemic forecast^56^ due to their acute capability to learn time series. Among these methods, uni-variate^57^ and multi-variate^58,59^ LSTM models have been successful at predicting influenza and COVID-19, mainly due to their capability to memorize long-term dependencies. DeepGLEAM^60^ uses a stochastic Diffusion Convolutional RNN (DCRNN) model, which considers short (commuting) and long range (air flight) mobility network connections, to forecast COVID-19 deaths at county and state granularities. Other versions of recurrent neural networks, such as attention networks^61^ and bidirectional LSTM^62^ networks, have also been reported to be successful. Taking stock of best practices in the extant literature, we propose a multi-variate and multi-time series long short-term memory (MTS-LSTM) network to simultaneously forecast confirmed cases, deaths, and mobility of all sub-populations at the county level using a single model. A study predicting the COVID-19 pandemic at this local level would provide unique insights for the policymakers to make targeted interventions to cope with the pandemic, given that many critical operational decisions and actions are made locally.

Previous models of epidemics have used a variety of covariates including mobility, underlying health, socio-demographic and socio-economic variables for predicting COVID-19 confirmed cases and death counts. In line with this precedence, we started with the implementation of models with various sets of independent variables. These variables included population density and measures at the sub-population level of race, educational attainment, age, family size and status, poverty, income, employment, housing, being in metropolitan or micropolitan area, health insurance status, percentage of deaths from other health conditions, percentage change in the time spent at work from Google mobility reports^63^, and number of visits to Points of Interest from SafeGraph Places Schema data sets^64^. After training a whole suite of model specifications and investigating the impact of these variables on pandemic severity by comparison of respective Root Mean Square Error (RMSE) statistics, it was found that COVID-19 is predicted with the highest accuracy with the SafeGraph mobility data as the sole covariate feature. The capability of the proposed deep learning model to predict COVID-19 dynamics with high performance with a single covariate is one of its distinctive strengths.

## Data pre-processing

In this research, we proposed a multi-variate recurrent neural network with LSTM layers to predict the dynamics of a pandemic, which learns from multiple sample time-series simultaneously. To assess the performance of the proposed multi-time series long short-term memory (MTS-LSTM) method, we collected data on COVID-19 confirmed cases and deaths and foot traffic at the county level for a period of 33 weeks between January 26th and September 12th, 2020.

First, the data for deaths and confirmed cases of COVID-19 infection were downloaded from the Center for Systems Science and Engineering (CSSE) at Johns Hopkins University^65^ and pre-processed to correct inaccuracies. During data cleaning, for example, instances of decrease in cumulative counts were identified and replaced by the value of the previous day. We calculated the weekly sum of confirmed cases and deaths corresponding to the Morbidity and Mortality Weekly Report (MMWR) weeks used by the Centers for Disease Control and Prevention (CDC) for all counties in lower 48 states of the US. MMWR weeks are based on the epidemiological calendar, with start on Sunday and end on Saturday. Daily reported cases and deaths were not selected since the new cases data are typically noisy. Instead, one may use the daily moving average. However, it is important to ensure no data leakage occurs between training, validation, and test data sets due to temporal dependency.

Next, the daily foot traffic patterns and points of interest (POI) for the top 5500 national brands were obtained from SafeGraph’s Places Schema data set^64^. SafeGraph provides visit data from around 18 million unique devices nationwide, which translates to 5–6% of the US population. These data are provided freely upon request for research purposes through the SafeGraph COVID-19 Data Consortium. We pre-processed and aggregated the foot traffic of the POIs into the county geographies and calculated their trailing average for each week.

Finally, to evaluate our model, we obtained predictions of an ensemble model implemented by COVID-19 Forecast Hub in collaboration with CDC for the period of 4 weeks ending on September 12th, 2020. Every week, the ensemble model accepts forecasts of COVID-19 confirmed cases and deaths from eligible models with a variety of assumptions and methodologies and takes the arithmetic mean and median of the forecasts for each geographic location. Evaluating the performance of the ensemble model and comparing it with the performance of the series of input models, the COVID-19 Forecast Hub recommends using the ensemble instead of individual models for policy considerations^66^. In addition, since the ensemble model is an average of several input models, it has less variability in performance over time than a single input model. As a result, the ensemble model is a good criterion for assessing the performance of our model. However, only predictions of new cases are available for the ensemble model.

## Model implementation

The codes were written in Python using the TensorFlow 2.5.0 package for deep learning. The implementation was conducted in the Google Colaboratory environment with GPU accelerators and high-RAM run-time shape. The model predicts all variables at the same time with a 4-week horizon (T = 4) and a time step of 1 week. We had confirmed cases, deaths, and mobility data for 33 weeks. The time series were divided into three sets. The last 4 weeks of the time series for all counties were kept aside for the test. We did not use data from these 4 weeks in any instances of the training process. Predictions of the Ensemble Model for the same 4 weeks were downloaded for evaluating the MTS-LSTM. Then, we used an out-of-sample validation (cross-validation) for training our model on the remaining 29 weeks (n = 33 − 4 = 29). After pre-processing of the time series, as explained in the model section, 70% of the feature vectors were used for training, and the rest (30%) for validation. This process was repeated ten times, each with a new set of randomly selected training and validation sets. All models in our experiments were trained using a batch size of 1024, an Adam optimizer with an initial learning rate of $10^{-3}$ and Huber loss function for 100 epochs. After a set of experiments, the window size was optimized with a length of three time steps (l = 3). This agrees with findings from other research that show the number of daily new cases of COVID-19 is related to population mobility of 3 weeks prior^67^.

## Results and discussion

Our research had three specific objectives. First, we aimed to study the impact of adding foot traffic (mobility) on prediction performance. Then, we wanted to find out how using multiple sample time series affects the performance of the predictions. Finally, we compared our predictions with the ensemble model.

To address the first question, we implemented the model with two specifications. The first model includes time series of two variables, deaths and new cases (m = 2), while the second model also includes the foot traffic as the third variable (m = 3). For the second objective, we trained the two models with different fractions of counties (1%–5%–10%–25%–50%–100%), henceforth called “experiments”, randomly selected among all counties in the data set and involved in the training and validation processes. We trained the model with ten “replications” to estimate the variation of predictions, each with a different set of randomly selected counties using a Monte Carlo sampling method.

**Figure 3:**
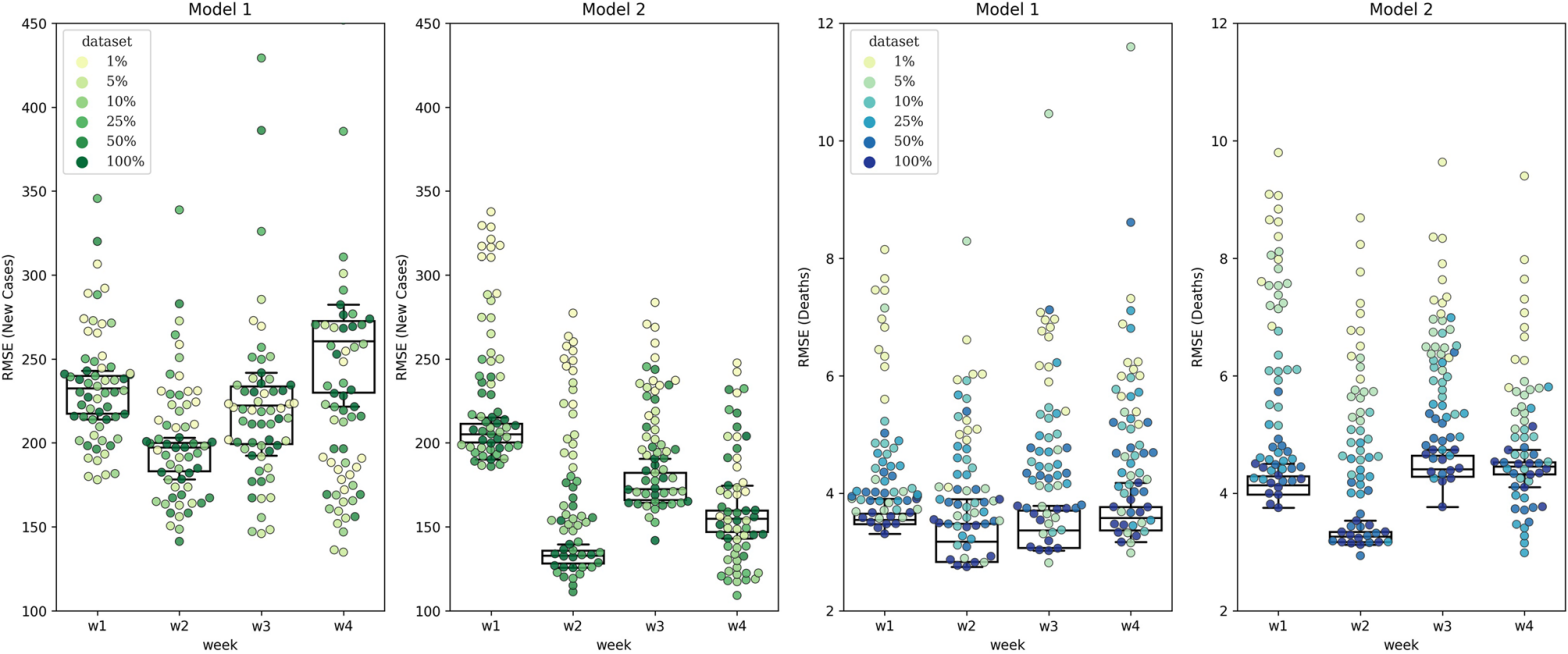
Comparison of predictions of Model 1 (without mobility as a predictor) and Model 2 (with mobility as a predictor). Each color represents a specific “experiment”, and each point represents a “replication” in the Monte Carlo process. The box-and-whisker diagrams are plotted based on the forecasts from the experiment with 100% of counties.

Results are presented in Fig. 3, where the two panels on the left show the ${\text{RMSE}}_{\text{week}}$ of new infection cases for all counties, and the two panels on the right show the same measure for deaths in all counties. Colors represent the portion of counties that have been used in an experiment (1 to 100%). The darker the color, the larger the fraction of counties that has been used in the experiment. Each observation point represents prediction of a single replication from the Monte Carlo process. In other words, points with the same color show all replications of a specific experiment. Notice that the Box-and-Whisker diagrams are plotted based on the forecasts from the experiment with 100% of counties.

What stands out in this figure is that Model 2 (with mobility as a predictor) predicts the new cases with lower RMSE median and variance than Model 1 (without mobility as a predictor) when 100% of counties are used in the training processes, and the same can also be said for experiments with smaller fractions of counties. Interestingly, Model 1 shows smaller RMSE values for the deaths compared to Model 2, while the differences are not significant. One possible explanation for this is that the window size is optimized with respect to the relationship between mobility and new cases. At the same time, the number of deaths has an extra delay in response to the number of new cases. That is, training the second model with a larger window size (possibly five or six for COVID-19) may lead to smaller death RMSE values for the second model compared to the first. From the figure, we can also find out that involving population mobility in the model improves the performance for predicting over a longer horizon window. Predictions of new cases for the third and fourth weeks have RMSEs less than the first-week prediction RMSE in the second model and their variance remains low. Median performances in the prediction of deaths are rather similar with the two model over a longer horizon, but Model 2 exhibits lower variances.

The color-coded RMSE values under different experiments show how increasing the number of counties in the training process affects the median and variance of the predictions (in Fig. 3). The pattern is more consistent with the death predictions mainly because the data on new infection cases are more subject to noise in comparison to the death data, which is more reliable.

The MTS-LSTM network may result in small negative predictions for some counties. For the confirmed cases for example the minimum value for 10 Monte Carlo repetitions is − 9.076, which is very small. The other two variables always remain positive in predictions. All negative values are replaced by zero before calculating RMSE values for evaluation. Alternatively, one could add an activation function to the last dense layer to make sure all predictions are non-negative.

**Figure 4:**
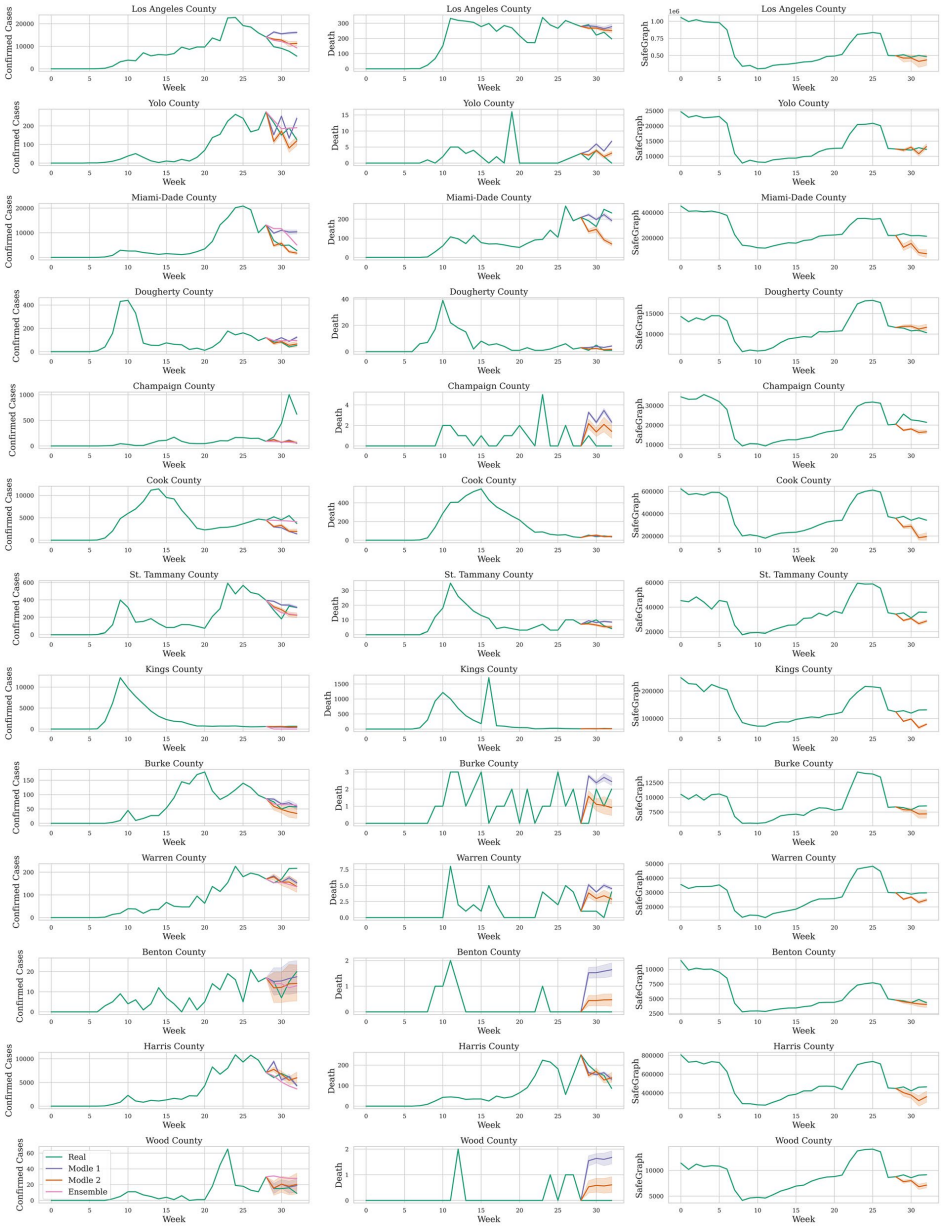
Dynamics of the new cases (left), deaths (middle), and foot traffic (right) over 33 weeks between January 26th and September 12th, 2020. Last 4 weeks include predictions from Model 1, Model 2, and the ensemble model and 95% intervals of confidence. Prediction of deaths is not available with the ensemble model.

Finally, we compared the prediction results of models 1 and 2 with the ensemble model from CDC. The ensemble model predictions for the 4 weeks between August 17th and September 12th were obtained^68^. Figure 4 shows the dynamics of the new cases (left), deaths (middle), and foot traffic (right) over 33 weeks for several indicative counties. The predictions for the last 4 weeks from all three models are also reported in the panels of this figure. Models 1 and 2 are represented with 95% confidence intervals. Predictions based on the ensemble model are point predictions. Most counties have experienced the same mobility pattern over the study period, while their deaths and new cases series are shown to follow different trends and to be in different stages across the study period, including during the prediction horizon window. Additional results for counties with large, medium or small population size are available in the supplementary materials (see additional information section). These illustrative cases suggest that predictions from our MTS-LSTM model are overall acceptable, but that great variability exists across different population communities in the US.

**Figure 5:**
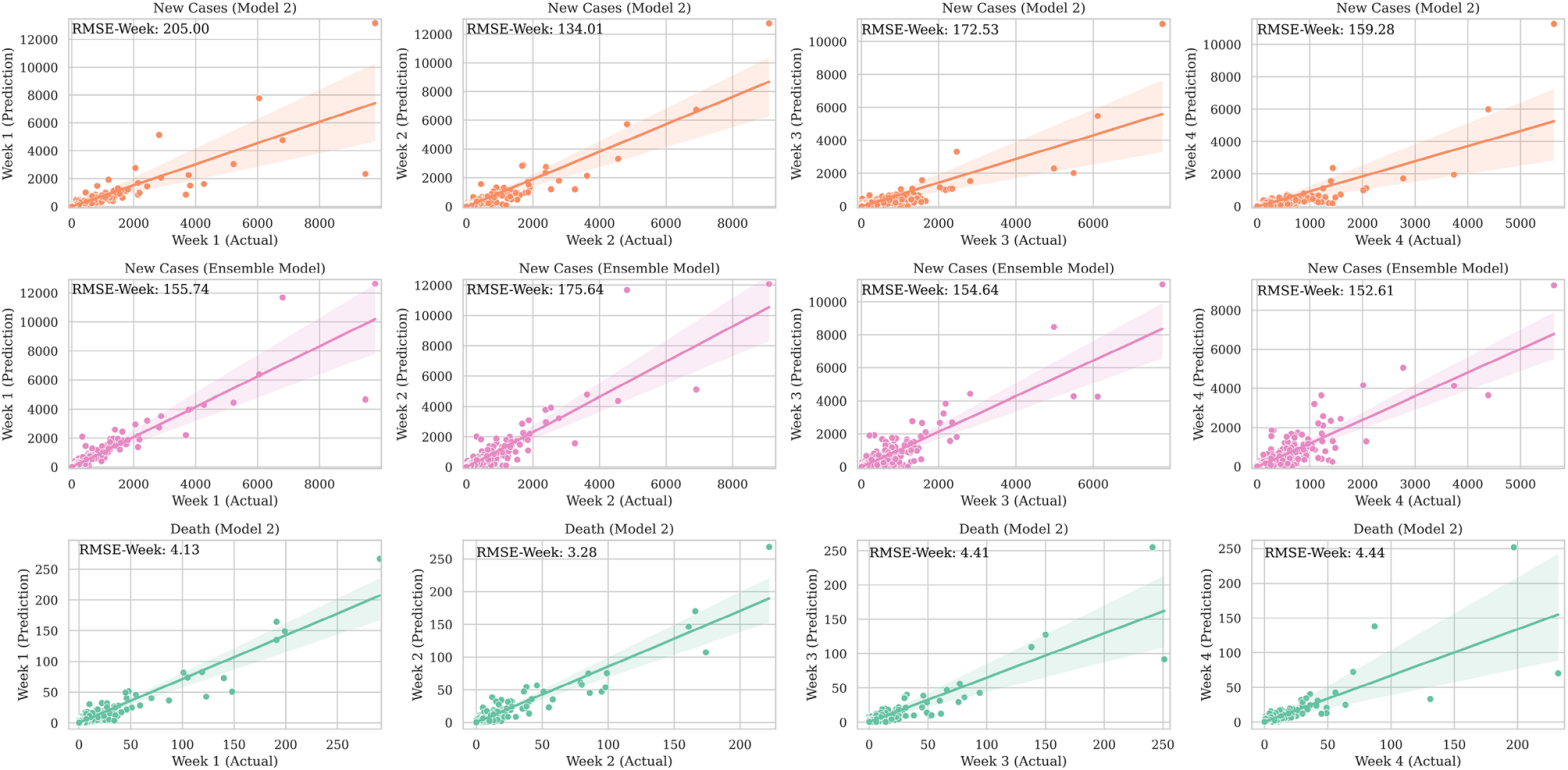
Predicted vs. real observations for new cases in Model 2 (top), new cases in ensemble model (middle), and deaths in Model 2.

**Figure 6:**
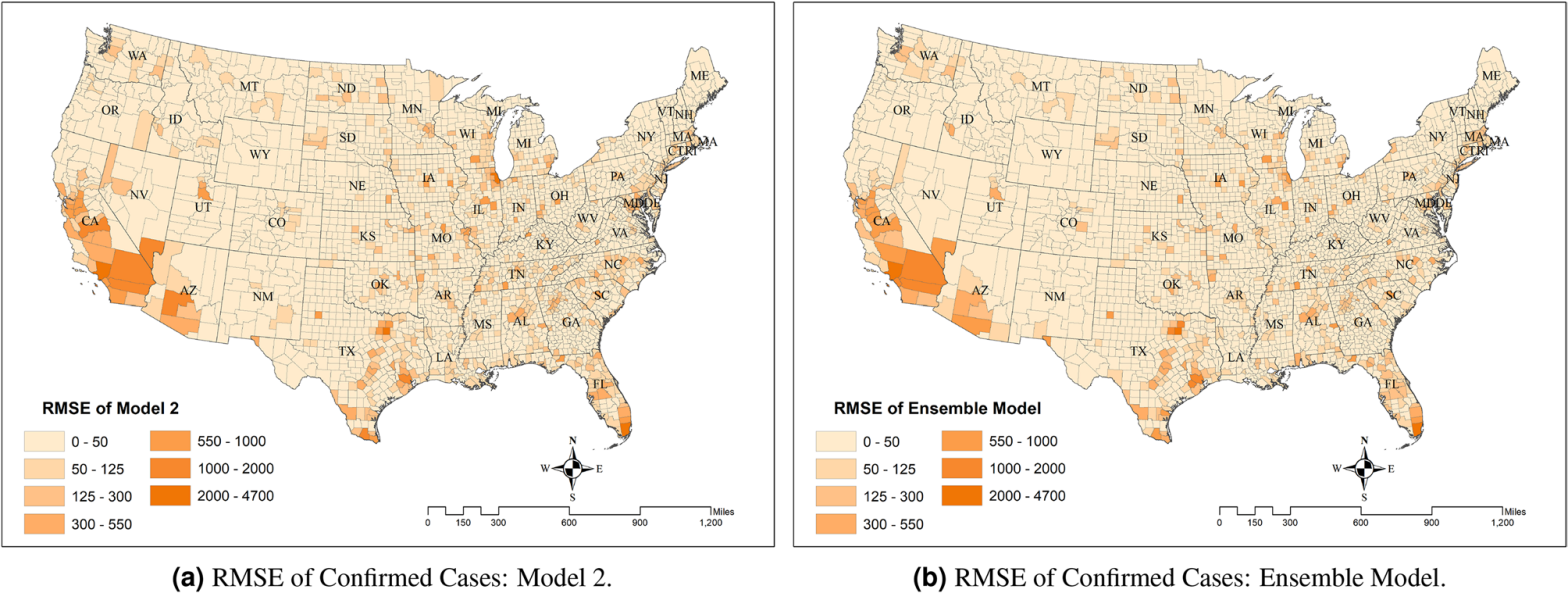
Comparison of predictions from Model 2 and Ensemble Model.

A deeper comparison between predictions from Model 2 and the ensemble model is available in Fig. 5, where predicted values for each county are plotted against their actual values for each model by week of the prediction horizon. Rows one and two represent the new infection cases from Model 1 and new cases from the ensemble model, respectively. The ${\text{RMSE}}_{\text{week}}$ values for the two models are in the same range, indicating very similar performance between the two models. This can also be seen in the two maps of Fig. 6, where the ${\text{RMSE}}_{\text{county}}$ measure is depicted for both models. The third row in Fig. 5 shows the death prediction results against their actually reported values. As we observed in Fig. 3, the model’s performance to predict deaths is higher than for new cases, which is an indication that the deaths data are more reliable than data on new cases. ${\text{RMSE}}_{\text{total}}^{\text{new cases}}$is equal to 224.28, 169.84, and 159.92 for Model 1, Model 2, and the ensemble model, respectively. ${\text{RMSE}}_{\text{total}}^{\text{deaths}}$ for Model 1 and Model 2 equals to 3.44 and 4.09, respectively.

## Multi-time series long short-term memory model

We designed a multi-variate recurrent neural network with LSTM layers to predict three variables of COVID-19 confirmed cases and deaths, as well as foot traffic for multiple sub-populations using an iterated multi-step (IMS) estimation. LSTM is a gradient-based method developed by^69^ to efficiently learn to store information over extended time intervals by recurrent back-propagation. This information is the long-term dependencies in a time series and can be transferred using a structure called memory cell. Memory cells have three gate structures named, input, forget, and output gates. These gates control the usage of historical information^70^. Several versions of LSTM have been developed for applications in disease spread^59,60^.

To aid the presentation of model results, we may refer to a uni-variate time series as a “feature”, and to a multi-variate time series as “features” from time-to-time. Also, a “feature vector” represents a sub-sequence of a multi-variate time series. Batches of feature vectors are the inputs to the network.

### Data model preparation

**Figure 7:**
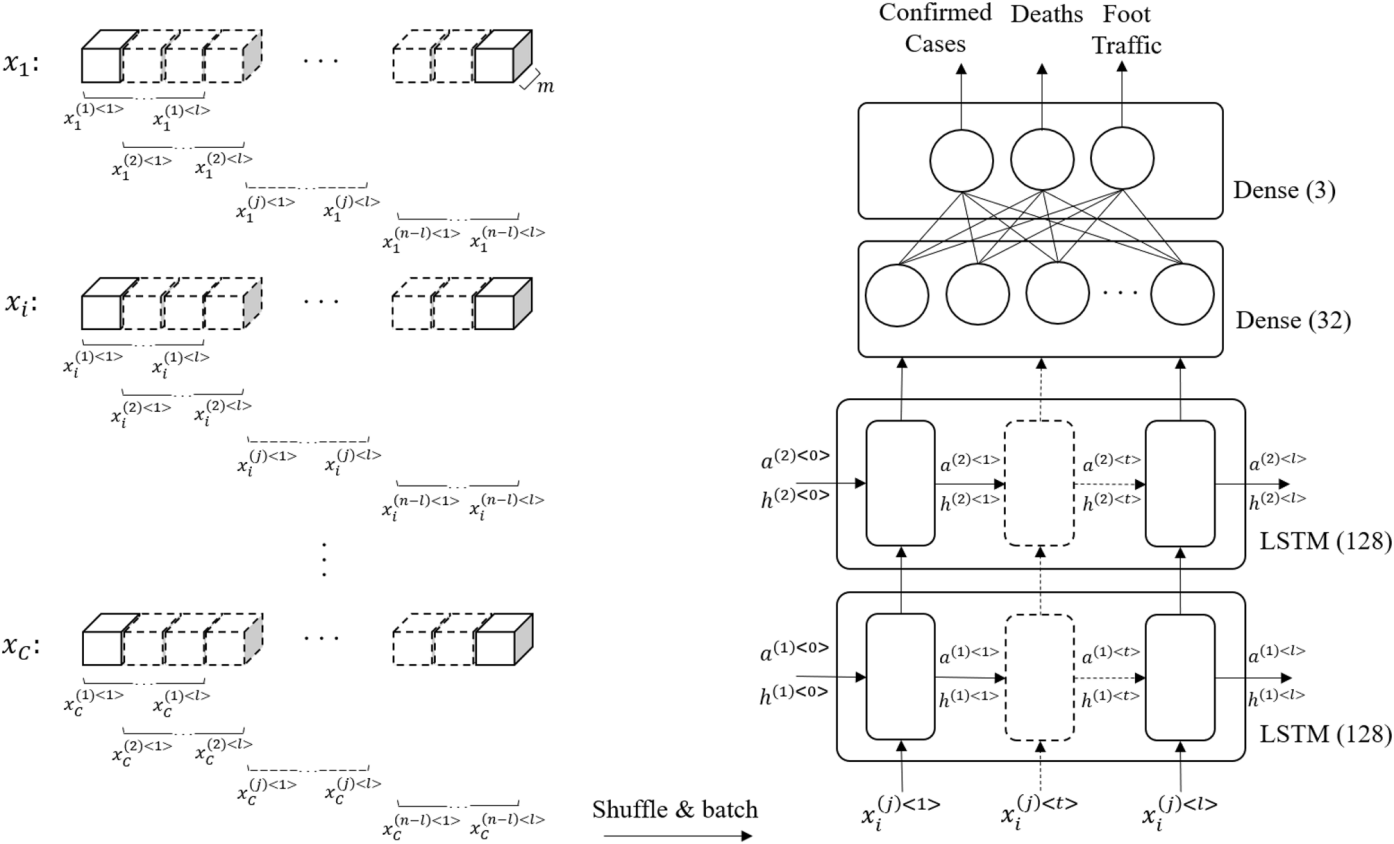
Proposed MTS-LSTM Model.

Figure 7 demonstrates the data preparation and architecture of the network. $\text{x}_\text{i}$ is a multi-variate time series for sub-population $\text{i}$ with $\text{n}$ time steps and $\text{m}$ features. A window $\text{L}$ of length $\text{l}$ is selected to create the feature vectors and labels from each time series. $\text{x}_\text{i}^{(\text{j})<\text{t}>}$ represents the time-step $\text{t}$ of feature vector $\text{j}$ from sub-population $\text{i}$. The sequence of feature values in each window (feature vector) is used to predict the next time step, where the actual values of the time series for those steps are used as the labels. In the process of creating feature vectors from each time series, the last sub-sequences with lengths less than $\text{l}$ are dropped. This means that each time series has $\text{n}-\text{l}$ feature vectors of the same length.

We shuffled feature vectors from time series of all sub-populations to create a bag of feature vectors. Such an approach has three advantages compared to the single time series forecasting with LSTM layers. First and foremost, it provides more data for training compared to using the time series of each sub-population separately. Data are very limited at the early stages of the outbreaks, both in frequency and in quality. Therefore, data-driven approaches, especially deep learning methods with many parameters, are not applicable or end up with inaccurate predictions. Second, the spread of infectious diseases such as COVID-19 has complex diffusion and relocation processes following mobility patterns across space and time^71^. That is, disease spread is not uniform, and outbreaks are in different stages in sub-populations across space. Such a method of creating a bag of feature vectors from all sub-populations provides an opportunity for the network to learn from later stages of an outbreak in sub-population A and uses this to predict outbreak dynamics in sub-population B. Third, training the machine learning model on feature vectors of multiple sub-populations simultaneously is expected to have more robust predictions when predicting disease dynamics spread in a new sub-population C. At the same time, intrinsic characteristics of different sub-populations such as mobility can affect the dynamics of disease in sub-populations. Thus, it is useful to have a multi-variate model that takes into account the mobility of sub-populations. In the next step, batches (bags) of feature vectors are created as inputs to the network. The size of each batch is klm, where k is the number of feature vectors within each batch.

### MTS-LSTM architecture

The network has two LSTM layers with 128 neurons each, followed by a fully connected layer of 32 neurons, a drop out layer, and a fully connected regression layer with m neurons representing m features to be predicted. In our MTS-LSTM model, we have three features: new cases, deaths, and foot traffic. The structure of an LSTM memory cell is represented by the following equations:

1 $${\tilde{\text{h}}}^{<\text{t}>} =   {\tanh}(\text{W}_\text{h}[\text{a}^{<\text{t}-\text{1}>},\text{x}_\text{i}^{(\text{j})<\text{t}>}]+\text{b}_\text{h})$$

2 $$\begin{aligned} \Gamma _\text{u}= \sigma (\mathrm{W}_\text{u}[\mathrm{a}^{<\mathrm{t}-\mathrm{1}>},\mathrm{x}_\text{i}^{(\mathrm{j})<\mathrm{t}>}]+\mathrm{b}_\text{u})\end{aligned}$$

3 $$\begin{aligned} \Gamma _\text{f}=  \sigma (\mathrm{W}_\text{f}[\mathrm{a}^{<\mathrm{t}-\mathrm{1}>},\mathrm{x}_\text{i}^{(\mathrm{j})<\mathrm{t}>}]+\mathrm{b}_\text{f})\end{aligned}$$

4 $$\begin{aligned} \Gamma _\text{o}= \sigma (\mathrm{W}_\text{o}[\mathrm{a}^{<\mathrm{t}-\mathrm{1}>},\mathrm{x}_\text{i}^{(\mathrm{j})<\mathrm{t}>}]+\mathrm{b}_\text{o})\end{aligned}$$

5 $$\begin{aligned} \text{h}^{<\text{t}>}=  \Gamma _\text{u}*{{\tilde {\text{h}}}}^{<\text{t}>}+\Gamma _\text{f}*\text{h}^{<\text{t}-\text{1}>}\end{aligned}$$

6 $$\begin{aligned} \mathrm{a}^{<\mathrm{t}>}= \Gamma _\text{o}*\mathrm{tanh}(\mathrm{h}^{<\mathrm{t}>}) \end{aligned}$$

where ${\tilde{\mathrm{h}}}^{<\mathrm{t}>}$ is the candidate value for updating memory cell parameter $\mathrm{h}^{<\mathrm{t}>}$. $\Gamma _\text{u}$, $\Gamma _\text{f}$, and $\Gamma _\text{o}$ are the update, forget, and the output gates. $\mathrm{a}^{<\mathrm{t}-\mathrm{1}>}$ is the activation value from time step $\mathrm{t}-\mathrm{1}$ of the feature vector. $\mathrm{W}_\text{h}$, $\mathrm{W}_\text{u}$, $\mathrm{W}_\text{f}$, and $\mathrm{W}_\text{o}$ are the network parameters and $\mathrm{b}_\text{h}$, $\mathrm{b}_\text{u}$, $\mathrm{b}_\text{f}$, and $\mathrm{b}_\text{o}$ are the biases.

The output features from the last LSTM layer, which provide representation for cases, deaths, and mobility, are concatenated before entering to the dense layer to provide a new feature representation. This new feature has information from previous time steps (time lags) of the feature being predicted as well as previous time steps (time lags) of secondary features. This way, all temporal dependencies, including various time lags between features, implicitly contributes to the predicted value of a feature in the back-propagation process. This is the simplest way to exploit temporal dependencies of various features. More explicit ways, with an added layer of complexity, exist to exploit dependencies of various features. For example, the cross-modal (cross-feature) LSTM^72^ separates the input features into a single time series and passes each of those through a separate three-layer LSTM stream and can be used for this purpose. A mechanism for information flow between the LSTM streams is proposed by authors to explicitly exploit multi-feature dependencies. The outputs from these LSTM layers are then concatenated into a new feature representation. This approach is more explicit because new hyper-parameters are added to the model that can be controlled in a fine-tuning process. In this research study, we use the former approach. However, it is of interest to compare these two approaches of modeling between feature temporal dependencies in the future research.

Finally, one may use either of two approaches to forecast variables for multi-steps^73^. The first approach is to optimize the multi-step forecasting objective function directly. This method is called direct multi-step (DMS) estimation, which is usually computationally expensive. Furthermore, the optimization of the objective function may fail if the model is highly non-linear or when the parameters are not well initialized^74^. The other approach is to predict step by step in an iterative way. This method is called iterated multi-step (IMS) approach. The choice of method depends on the bias and variance of the prediction, on the number of prediction steps, and on the non-linearity of the model^75^. IMS is easy to train, and forecasts of arbitrary length are possible. However, it is prone to accumulative errors^74^. In this research, we used an IMS method since the length of the prediction horizon is not long.

## Evaluation

We use three RMSE based metrics to evaluate predictions:

7 $$\begin{aligned} \mathrm{RMSE}_\text{county}=  \sqrt{\frac{\sum _{\mathrm{t}=1}^{\mathrm{T}}(\mathrm{P}_\text{t} - \mathrm{O}_\text{t})^2}{\mathrm{T}}} \end{aligned}$$

8 $$\begin{aligned} \mathrm{RMSE}_\text{week}= \sqrt{\frac{\sum _{\mathrm{j}=1}^{\mathrm{C}}(\mathrm{P}_\text{j} - \mathrm{O}_\text{j})^2}{\mathrm{C}}} \end{aligned}$$

9 $$\begin{aligned} \mathrm{RMSE}_\text{total}= & {} \sqrt{\frac{\sum _{\mathrm{j}=1}^{\mathrm{C}}\sum _{\mathrm{t}=1}^{\mathrm{T}}(\mathrm{P}_{\mathrm{jt}} - \mathrm{O}_{\mathrm{jt}})^2}{\mathrm{CT}}} \end{aligned}$$

$\mathrm{RMSE}_{\mathrm{county}}$ calculates the RMSE of multi-step predictions for a single sample (county). T is the length of the prediction horizon and $\mathrm{P}_\text{t}$ and $\mathrm{O}_\text{t}$ are the prediction and observation for time step t. $\mathrm{RMSE}_{\mathrm{week}}$ represents the RMSE of predictions for a single time step (week) and all counties, where j is the county index and C is the number of counties. Finally, $\mathrm{RMSE}_{\mathrm{total}}$ presents a single-value metric based on RMSE with all predictions in different counties and weeks.

## Conclusions and future work

We proposed a multi-variate LSTM-based recurrent neural network with mobility trained on multiple time series samples at the same time to predict the spatio-temporal spread of a pandemic. Our results show that adding mobility as a variable and using multiple samples to train the network improve the performance of the predictions both in terms of the bias and of the variance of forecasts. We also showed that the predicted results have similar accuracy and spatial patterns with the ensemble model used for benchmarking. While this may not add a new capability in modeling performance, our MTS-LSTM model has several attractive advantages. Compared to the single time-series LSTM, our model predicts the dynamics of disease within different sub-populations simultaneously. For a study area with n sub-populations, we used a single model instead of n different models. Given our experimental results, it may also be possible to replace the ensemble model which is based on several other models with a single model exhibiting the same level of performance, which would reduce data inputs and computational resources. From the point of view of training data size, this approach takes advantage of disease spread data from a sub-population to predict disease dynamics in other sub-populations. This is a very attractive feature, especially at the beginning of pandemics. At such crucial times, data availability is very limited, and machine learning and deep learning methods can be challenging to implement and use.

Recurrent neural networks are capable of being updated with new sets of observations as they become available. From this point of view, they are comparable with Bayesian learning. Besides, our model predicts all variables at the same time. This is important because of the causal relationship between variables that may impact the predictions. For example, staying at home when volunteering may be affected by the current number of new cases. Also, the model is highly flexible to add new variables such as hospital capacity or vaccination rates, as reliable data become available.

There is room to further improve our MTS-LSTM model. First, we optimized the size of the moving window based on the RMSE of new cases. It is better to optimize this hyperparameter by considering all variables (in our research, new infection cases and deaths). Second, we predicted the dynamics for a single period of time. For the full evaluation of the model, it is useful to use the current model to predict dynamics for other periods as well. Third, after using a specific amount of data (about 10%), the model starts to improve slowly. This means, on the one hand that, if we have data from a portion of counties, we can get to an acceptable level of accuracy to predict dynamics of disease for all counties. On the other hand, this means there is room to use more complex models. In this respect, experimenting with different architectures such as adding bidirectional LSTM layers, 1D convolutional layers, encoder-decoder layers, and attention layers, presents a frontier for future work with opportunity to further enhance the predictive power of the MTS-LSTM model. Fourth, efficacy may also be improved by explicitly modeling temporal dependencies between features using a Cross-modal (cross-feature) LSTM^72^. Fifth, we did not account for spatial dependencies between counties in this research. All counties that participate in predictions of dynamics for a county have the same weights. However, we expect the counties closer to each other to have more similar values and, as a result, higher weights. Thus, it is useful to apply methods to include the spatial dependence. This can be conducted in a preliminary process using geo-statistical models or using the integration of LSTM with convolutional neural networks. The integration of LSTM with convolutional layers in graph based neural networks is also an interesting line of future research for disease spread prediction^60,76,77^ . Along a somewhat related line of thought, our analysis did not support using a series of covariates as predictors, which would better differentiate between local conditions. We would conjecture, however, that this requires further study, especially in the context of a model structure that captures spatial relationships more comprehensively.

Finally, the model can also be extended to series at different scales and granularities (e.g., state level and county level) to establish how performance changes or to experiment with policy-related scenarios (e.g., statewide lockdowns) that operate at various jurisdictional levels. Our MTS-LSTM model was trained with COVID-19 cases and deaths series in the United States and for a specific time period. It will be important to use this pre-trained model to predict disease dynamics in other geographic contexts, time periods, or even similar infectious diseases such as influenza in order to demonstrate the reproducibility of our methodology and results.

## Data Availability

Supplementary Information (e.g., codes, data, results) are available in GitHub at https://github.com/behnamnkp/Covid-19-Prediction.git.
